# Point-of-Care Testing in PKU: A New ERA of Blood Phenylalanine Monitoring

**DOI:** 10.3390/nu17233800

**Published:** 2025-12-04

**Authors:** Alex Pinto, Adam Gerrard, Suresh Vijay, Sharon Evans, Anne Daly, Catherine Ashmore, Maria Inês Gama, Júlio César Rocha, Rani Singh, Richard Jackson, Anita MacDonald

**Affiliations:** 1 Birmingham Children’s Hospital, NHS Trust, Steelhouse Lane, Birmingham B4 6NH, UK; adam.gerrard@nhs.net (A.G.); suresh.vijay1@nhs.net (S.V.); sharon.morris6@nhs.net (S.E.); a.daly3@nhs.net (A.D.); catherine.ashmore@nhs.net (C.A.); maria.gama1@nhs.net (M.I.G.); anita.macdonald@nhs.net (A.M.); 2 Nutrition and Metabolism, NOVA Medical School (NMS), Faculdade de Ciências Médicas (FCM), Universidade Nova de Lisboa, 1169-056 Lisboa, Portugal; rochajc@nms.unl.pt; 3 CINTESIS@RISE, Nutrition and Metabolism, NOVA Medical School (NMS), Faculdade de Ciências Médicas (FCM), Universidade Nova de Lisboa, 1169-056 Lisboa, Portugal; 4 Reference Centre of Inherited Metabolic Disease, Unidade Local de Saúde São José, 1069-045 Lisboa, Portugal; 5 Comprehensive Health Research Centre (CHRC), NOVA Medical School (NMS), Faculdade de Ciências Médicas NMS/FCM, Universidade Nova de Lisboa, 1169-056 Lisbon, Portugal; 6 Department of Human Genetics, Emory University School of Medicine, Atlanta, GA 30322, USA; rsingh@emory.edu; 7 Department of Health Data Science, University of Liverpool, Liverpool L69 3GJ, UK; richj23@liverpool.ac.uk

**Keywords:** point-of-care, home monitoring, phenylalanine, phenylketonuria

## Abstract

**Background:** In phenylketonuria (PKU) patients, dried blood spot (DBS) sampling remains the standard method for monitoring phenylalanine (Phe) levels. However, delays in reporting results can hinder timely dietary adjustments. Patients and caregivers have expressed a preference for point-of-care testing (POCT) devices that enable home-based monitoring. **Objectives:** Our aim was to compare blood Phe measurements in PKU patients and caregiver usability of a POCT system with DBS, which is the standard practice monitoring method. **Methods:** Twenty participants (eighteen children with PKU and two healthy controls) were recruited. Caregivers of children with PKU were asked to perform blood Phe measurements at home under the supervision of a researcher, using both the POCT device (Egoo Phe system) and DBS sampling. Healthy controls collected the same number of samples using both methods in a hospital setting. The POCT system required 40 µL of blood and used an enzymatic, bioluminescent detection system. DBS samples were analyzed by tandem mass spectrometry (TMS) and required two blood spots (approximately 100 µL of blood). The Egoo Connect App, linked via Bluetooth to the POCT device, displayed results after 29 min. Caregiver usability of the POCT system was assessed using questionnaires at each visit. **Results:** A total of 100 paired samples were collected. Median values were 274 μmol/L (range: 30–1039) for POCT and 270 μmol/L (range: 20–1190) for DBS. POCT readings were a mean of 4.6% higher than DBS with a noticeable strong correlation observed (y = 1.017x; R^2^ = 0.8450; *p* < 0.0001). The usability of the POCT system improved with caregiver practice, and all caregivers expressed a preference for POCT over DBS. **Conclusions:** The POCT system for blood Phe demonstrated strong concordance with DBS and high caregiver acceptance, highlighting its potential to transform PKU care through faster, patient-driven monitoring and more timely clinical decision-making.

## 1. Introduction

Phenylketonuria (PKU) is an autosomal-recessive disorder characterized by phenylalanine hydrozylase (PAH) deficiency encoded by the PAH gene. It causes elevated levels of phenylalanine (Phe) in the blood, brain, and other tissues, resulting in neurocognitive impairment if left untreated [1]. PKU is detected by neonatal screening and requires lifelong treatment. Though the condition is primarily managed with a Phe-restricted diet and supplemented with free/low-Phe protein substitutes, a subset of individuals may also be prescribed adjunct pharmaceutical treatments, including sapropterin, sepiapterin, and pegvaliase, to reduce blood Phe levels and consequently prevent adverse neurological sequelae [2,3]. In PKU, blood Phe correlates strongly with Phe in the brain, cerebrospinal fluid, neurocognitive and behavioural outcomes and Phe levels are the principal biomarker for assessing metabolic control [4]. According to the European PKU guidelines, target blood Phe levels should be maintained at 120 to 360 µmol/L in children up to 12 years of age, and 120 to 600 µmol/L in individuals 12 years and over [4].

Accurate and reliable monitoring of blood Phe is central to the effective management of individuals with PKU. Blood Phe measurements are often taken less frequently than the European PKU guidelines recommends, and the frequency seems to deteriorate with age. Evidence indicates that weekly Phe testing is associated with improved metabolic control across all age groups, compared to less frequent monitoring [5]. This proactive approach enables timely dietary and therapeutic adjustments, helping to maintain Phe concentrations within recommended target ranges. In routine clinical practice, universal monitoring is conducted via dried blood spot (DBS) sampling. Caregivers and individuals with PKU typically perform DBS collection at home using a non-volumetric capillary blood sample obtained from a finger-prick (or from the heel in babies). Two single hanging drops of blood, sufficient to fill two pre-printed circles, are applied to filter paper and allowed to air dry before being mailed to the hospital laboratory [6,7]. This technique is minimally invasive, requires only a small blood volume, and is both quick and user-friendly. Additional advantages include ambient temperature storage and transport [8]. In the UK, DBS Phe analysis is conducted by flow-injection analysis–tandem mass spectrometry (FIA-MS/MS). This offers a rapid turnaround and high sample throughput [9].

The drawback of using DBS for blood Phe monitoring is its imprecision, with many pre, real time and post-analytical variables affecting the final test results [7]. High variability in DBS results is linked to inconsistencies in blood spot size and volume, and variable extraction recoveries [7,10,11,12,13]. Falsely low blood Phe is seen with small DBS < 8 mm in diameter (up to 35%), in compressed samples (up to 35%), [14,15,16] whilst multilayered specimens result in elevated results (elevated by up to 15%) [14]. Inadequate drying of DBS, high temperatures, high humidity, and contamination of the filter papers affect analyte results [7,16]. Other factors contributing to variation include the type of filtration paper, punch size and location, and extraction efficiency [10]. Elevated patient hematocrit levels may lead to higher blood Phe concentrations as whole blood samples with a higher haematocrit increase blood viscosity, tending to show less widespread distribution across the filter paper, and consequently the target analyte diffusion distance is shorter [12,17,18]. Moat et al. has estimated that a typical specimen containing 360 µmol/L of Phe could vary from 300 to 400 µmol/L in DBS using a standard 3.2 mm sub-punch [16]. Complementing this, a UK-wide study involving 10 laboratories analyzed 2111 DBS specimens from 1094 individuals with PKU [19]. Although only 4% of samples were formally rejected, the median rejection rate would have risen to 21.9% had strict DBS quality control criteria been applied. The majority of unacceptable samples were either too small or multi-spotted, highlighting the need for clearer guidance, caregiver training, and harmonized rejection thresholds across laboratories [19].

DBS is used as a proxy for venous sampling, although results are not directly comparable. While venous sampling is impractical for routine monitoring, DBS-derived Phe levels are typically 15–28% lower than plasma values due to matrix effects and sample variability [16,20,21,22,23] (Table 1). In contrast, Van Vliet et al. [24] reported comparable results between DBS and venous samples using high-performance liquid chromatography (HPLC) and FIA-MS/MS. Discrepancies between results are particularly pronounced in laboratories that do not apply a correction factor to align DBS concentrations with plasma equivalents.

Therefore, without rigorous quality control, DBS sampling may underestimate or misrepresent true blood Phe levels in PKU, which may impact clinical decision-making. The potential utility of point-of-care testing (POCT) for blood Phe monitoring has long been recognized by patients and caregivers. Surveys conducted within the PKU community consistently highlight a desire for more accessible and timely feedback mechanisms. In a recent study examining patient satisfaction of current home blood Phe monitoring methods, Kuypers et al. [25] reported that the majority of respondents believed home monitoring would facilitate better disease management, describing current systems as limited due to being time-consuming and logistically burdensome. If analytical reliability can be ensured, home monitoring may reduce pressure on hospital laboratory services, lower healthcare costs when used within appropriate clinical pathways, improve the speed of results, provide convenience, and lead to better patient empowerment and engagement [26]. These findings support the case for integrating validated home monitoring technologies into routine PKU management, provided that standardization, training, and quality assurance frameworks are in place.

Generally, rapidly developing healthcare technology and changes in clinical practice mean that the use of increasingly complex medical devices that allow patients to monitor their own blood markers is being encouraged by healthcare commissioning services [27]. In diabetes, POCT has demonstrated a positive impact on clinical workflows and patient management. Multiple studies have demonstrated that frequent self-monitoring of blood glucose significantly improves glycemic control in individuals with both type 1 and type 2 diabetes [28,29]. By comparison, continuous glucose monitoring (CGM) is also being used in Glycogen Storage Diseases (GSDs), a group of inherited metabolic disorders (IMDs). In GSDs, CGM is not used to guide insulin therapy but rather to detect and prevent hypoglycemia, particularly nocturnal and postprandial episodes that are often missed during intermittent blood glucose testing. Several studies have demonstrated that CGM improves symptom control, supports dietary optimization (e.g., cornstarch dosing and meal timing), and contributes to better long-term outcomes [30].

The Egoo Phe system is a novel POCT device developed for home use by individuals with PKU and their caregivers. It utilizes an enzymatic assay coupled with a bioluminescent detection system, converting light signals from a capillary blood sample into a quantitative Phe measurement. Results are available within approximately 29 min, providing a rapid and user-friendly alternative to conventional laboratory-based methods [31]. The primary objective of the present study was to compare blood Phe concentrations obtained via the Egoo system with those measured using the standard DBS method and analyzed by FIA-TMS employed at Birmingham Children’s Hospital. This longitudinal, controlled comparative study aimed to evaluate the accuracy, reliability, and caregiver usability of the Egoo system in routine PKU monitoring when operated by caregivers in a home setting.

## 2. Materials and Methods

### 2.1. Egoo Phe System (Version 2)

The Egoo Phe system is a novel POCT device designed to quantitatively measure blood Phe concentrations in capillary blood. The system enables a Phe measurement range of 30–1500 µmol/L. An early version (Version 1) of the Egoo Phe system was previously tested during a PKU camp at Emory University, USA [31]. Insights from this initial deployment informed key refinements in usability and performance, culminating in the development of Version 2, which was used in the present study.

The Egoo Phe system comprises four integrated elements:

- Egoo P300 Analyzer: A CE-marked device that performs the biochemical analysis.
- Egoo Phe Test Capsule: A capsule that contains reagents for Phe quantification.
- Egoo Collect: A plasma separation device that isolates plasma from whole blood.
- Egoo Connect App: A Bluetooth-enabled smartphone application that operates the analyzer, displays test progress, and delivers results in 29 min.

Blood is obtained via a heel or finger-prick. A pre-measured pipette is used to collect approximately 40 µL of blood, equivalent to two large blood droplets. The pipette is positioned above the Egoo Collect device and emptied with a single squeeze onto filter paper in the centre of the Egoo Collect. The Egoo Collect separates the plasma from the whole blood, which is then transferred into the Phe test capsule using a dedicated stick. The capsule is inserted into the Egoo P300 analyzer, initiating the test. The Egoo Collect is stable at room temperature (15 °C to 28 °C) but the Egoo Phe capsules must be stored at −15 °C to −25 °C in a commercial freezer. Capsules must be defrosted 29 min prior to use, and they remain stable for up to 72 h post-thaw [31].

**Figure 1:**
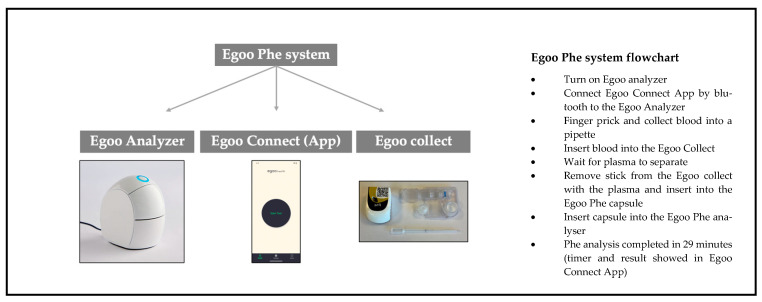
Description of the Egoo Phe POCT system.

To minimize participant burden, all equipment was stored and managed by the research team throughout the study period. A schematic of the Egoo Phe system is provided in Figure 1.

### 2.2. Calibration

The Egoo device was calibrated against the DBS reference method, and all POCT results were evaluated in comparison to DBS-derived values. Calibration of the Egoo device can be performed using several methods. For the purposes of this study, calibration was aligned with our laboratory’s established protocol, employing DBS analysis to ensure methodological comparability. Alternative calibration strategies could involve established reference standards, which may enable closer approximation to venous sample measurements.

Calibration standards were prepared and analyzed using the Biochrom amino acid analyser following standard laboratory procedures for plasma samples.

For the MS/MS method, standards were diluted and an equivalent of 3 µL was added to the internal standard volume typically used for a 3 mm blood spot punch (200 µL). The standards produced expected results, confirming that calibration across plasma-based and blood spot-based assays was consistent with the calibration of the Egoo Phe system.

Given the established bias between plasma and DBS Phe concentrations, a spiked-recovery experiment was conducted to assess calibration accuracy in whole blood. Whole blood was centrifuged to remove plasma, yielding packed red cells. Aliquots of 25 µL packed red cells were mixed with 25 µL of each calibrator level and applied in duplicate to DBS.

After air-drying for >3 h, 3 mm discs were punched and analyzed from each spot. For each calibrator level, the mean of duplicate measurements was calculated. The zero calibrator reflected the base Phe concentration of the packed red cells. All results were multiplied by two to correct for the 1:1 dilution with the calibrator. This enabled derivation of a linear calibration curve y = mx + c (where c = base Phe concentration). The calibration was verified using multiple Egoo Analyzers.

This method assumes a hematocrit of approximately 50%, supporting the 1:1 volumetric mixing of packed red cells with aqueous calibrators. However, individual variation in hematocrit may influence analyte distribution and recovery in DBS, introducing potential bias. Furthermore, the use of aqueous calibrators reduces sample viscosity, which may alter blood spreading dynamics on filter paper and affect spot morphology and homogeneity.

### 2.3. Routine Blood Phe Monitoring: DBS

Parents and caregivers were already trained and experienced in DBS collection. Healthy volunteers received structured training during their initial visit.

DBS samples were collected either at home (patient participants) or in a hospital setting (control participants) under the close supervision of trained researchers (A.M. and A.P.). DBSs were collected on filter cards, Perkin Elmer 226 (UK Standard NBS, Public Health England, London, UK). All the cards had a standard thickness and blood Phe was calculated on a 3.2 mm punch and quantified using FIA-MS/MS in accordance with validated protocols. Blood spots were returned to the laboratory at Birmingham Children’s Hospital directly by the research team and not by post in case of sample loss. They were analyzed within 48 h.

### 2.4. Patient Selection

All patients in the study were diagnosed with PKU by newborn screening, aged 3–17 years of age, on dietary treatment, ±sapropterin. Healthy adult volunteers, without PKU, were aged above 18 years.

Exclusion criteria included patients with comorbidities that may impact tolerance or the burden of blood sampling (e.g., autism or neurodiversity), patients with acute illness (e.g., chicken pox, tonsilitis requiring antibiotics) and patients with long-term chronic illnesses on long-term medications (e.g., diabetes). Children with PKU below 3 years or above 17 years of age were not included in the study.

### 2.5. Study Design

The study enrolled 18 children with PKU, aged 3 to 16 years, along with their caregivers, and 2 healthy adult volunteers. All participants were recruited from Birmingham Children’s Hospital. Healthy volunteers were included to ensure analysis of blood Phe levels below 100 µmol/L.

The study comprised five visits per participant, each separated by 7 days (±5 days) to accommodate scheduling flexibility and frequency of routine DBS monitoring.

- Study visits under home conditions were completed for all the participants with PKU. At each visit, two blood samples were collected:
  - One sample using the standard DBS method for routine monitoring.
  - One sample using the Egoo system (to assess usability and performance in a home setting).
- Healthy adult volunteers underwent the same sampling protocol, but all visits were conducted at Birmingham Children’s Hospital under supervised conditions. This allowed for controlled comparison of device performance and usability across different environments and user groups.

**Figure 2:**
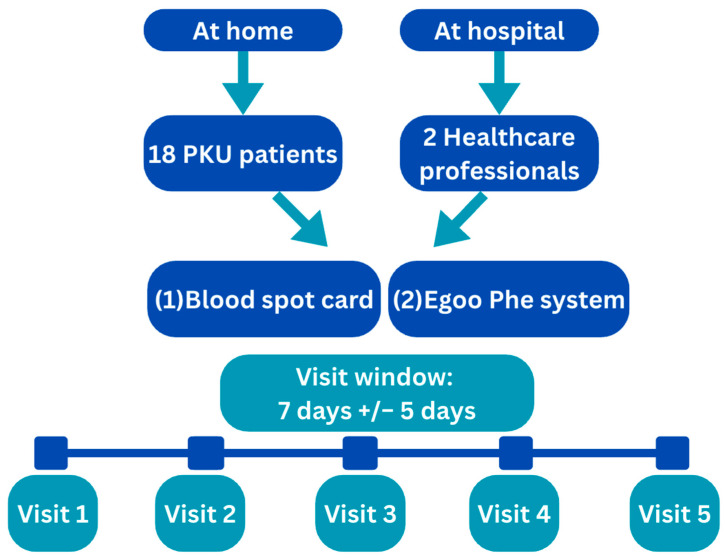
Study design.

Figure 2 shows the study design.

### 2.6. Training

All parents/caregivers of children with PKU and healthy adult volunteers received structured training on the use of the Egoo system during Visit 1. This session included (1) a verbal explanation of the device’s components and operation; (2) a demonstration of the system by a trained health professional; and (3) issue of a written visual instruction leaflet tailored for lay users. Each training session was brief, lasting approximately 10–15 min.

Across the subsequent four study visits, participants were offered reinforcement or refresher guidance as needed. This support was provided either proactively (if errors were observed) or upon participant request, ensuring consistent and confident use of the device in both home and clinical settings.

### 2.7. Procedure at Each Review

Participants were asked to perform the following procedures at each study visit.

- Obtain a DBS following standard procedure for blood Phe monitoring, i.e., approximately 100 μL blood collected as 2 hanging blood drops from a finger-prick that filled 2 circles on blood spot cards.
- Use the POCT Egoo system for blood sampling, i.e., collect 40 µL of capillary blood via a finger-prick into a pre-measured pipette. The blood sample was transferred to the Egoo Collect device for plasma separation, and then the sample was loaded into a dedicated Phe test capsule. The capsule was inserted into the Egoo P300 analyzer, which was activated via the Egoo Connect App. The blood Phe results were retrieved via the app.
- The blood samples were performed simultaneously (same date and time) for both methods.
- Both fasting (15%, n = 15/100) and non-fasting samples (85%, n = 85/100) were collected during the study.

### 2.8. Assessment of Usability and Participants’ Feedback on POCT for Blood Phe Monitoring

The usability and acceptability of the sampling methods were evaluated through structured questionnaires administered at each home visit (n = 5). Researchers conducted usability assessments during each study visit. Parents and caregivers completed a baseline questionnaire focused on the DBS method. At study completion, both parents/caregivers and healthy volunteers participated in a comparative evaluation of the DBS and Egoo Phe systems. Additionally, a post-testing survey was administered to assess user experience and perceived usability of the Egoo POCT system, capturing feedback from both caregivers and healthy volunteers.

Non-validated questionnaires were developed by the research team and reviewed to ensure that they captured each component of the process of performing blood Phe measurements using the POCT device.

### 2.9. Sample Size Calculation and Statistics

It was anticipated that blood Phe concentrations obtained via DBS sampling would be up to 30% lower than those measured by venous samples and the Egoo POCT. The calibration against DBS used in this study aimed to minimize this difference. Data were collected from 20 participants (18 patients and 2 healthy controls), each contributing five successive blood Phe measurements, yielding a total sample size of 100. This design ensured that a natural relative difference of 15% would produce a standard error of 1.5%, with a 95% confidence interval width of 5.88%. Blood Phe results across methods were compared using regression analysis techniques. Quantitative data and continuous variables were summarized using mean (±standard deviation [SD]) or median (range), while categorical variables were reported as absolute frequencies or percentages. All statistical analyses were conducted using GraphPad PRISM (Version 10.1.0, 18 October 2023; GraphPad Software, Boston, MA, USA), with statistical significance defined as *p* <  0.05. Passing Bablok regression was performed with R (Version 4.0).

### 2.10. Ethics

Parents/caregivers gave informed consent to participate in the study and age-appropriate assents were completed by participating patients. The “Declaration of Helsinki” (52nd WMA General Assembly, Edinburgh, Scotland, 3–7 October 2000) and Good Clinical Practice Guidelines were used in this study. Ethical approval was granted by the West-Midlands, Edgbaston ethical committee 20 March 2025 with the IRAS number 350589 and REC number 24/WM/0253. The study was registered with Clinicaltrials.gov with the ID NCT06940193.

## 3. Results

### 3.1. Participants

Twenty participants were recruited. Eighteen were caregivers/parents of children with PKU (eleven males and seven females) with a mean age of 8.3 ± 3.3 years (range: 4–14 y). Thirteen participants with PKU were all on dietary Phe restriction only, 1 was on sapropterin therapy only, and 4 were on a combination of dietary Phe restriction and sapropterin therapy. Two healthy adult volunteers (healthcare professionals) also participated in the study. They had a mean age of 36.5 ± 12.0 years (range: 28–45 y). The participant cohort included two individuals of Asian origin, nine Eastern Europeans, one Southern European, and eight of British origin. All patients completed all the study visits.

### 3.2. Baseline Questionnaire

This questionnaire was completed by caregivers of children with PKU (n = 18), all of whom had extensive experience of routine blood Phe monitoring. Since diagnosis, all caregivers had consistently (at least weekly) performed the finger-prick procedure for DBS sampling, demonstrating a reliable technique and familiarity with the process. The mean duration of caregiver-led blood sampling was 8.3 ± 3.3 years (range: 4–14 years), supporting a high level of procedural competence. From the questionnaire results, only 17% (n = 3 of 18) of caregivers waited one hour or more before posting their blood samples to the hospital, potentially resulting in compressed samples. Eighty-three percent (n = 15 of 18) had experienced sample rejection by the hospital laboratory, mostly associated with small blood spots (67%, n = 12 of 18). Thirty-three percent (n = 6 of 18) had experienced blood samples that had taken more than 14 days by the postal service to arrive in the hospital laboratory and were rejected for analysis. For n = 4 (22%) participants, this had occurred five or more times. Sixty-seven per cent (n = 12 of 18) of participants received their blood Phe results within 3 days.

Table 2 presents the results for the baseline assessment evaluating finger-prick blood spot tests performed by parents/caregivers.

### 3.3. Blood Phe Levels—Comparison Results Between POCT vs. DBS

Over five study visits, from 20 participants, a total of 100 paired blood samples were collected using both DBS and POCT via the Egoo system. Median Phe levels were comparable between methods: 274 µmol/L (range: 30–1039) for POCT and 270 µmol/L (range: 20–1109) for DBS. In the participants with PKU, 68% of POCT samples and 64% of DBS samples were ≤360 µmol/L. Ninety percent of POCT samples and 87% of DBS samples were ≤600 µmol/L. Supplementary Table S1 presents the proportion of samples below these thresholds across all visits.

Overall, POCT Phe levels were a median of 4.6% higher than DBS, with individual differences ranging from −77% to +190%.

Participant-reported challenges with the POCT procedure included air bubble formation within the blood pipette, incomplete sample transfer into the Egoo Collect filtration system, and inconsistent identification of the end-point signal indicating successful plasma separation.

One participant consistently showed lower Phe levels with POCT (31–52% below DBS), suggesting a possible individual specific anomaly. Full distributions of blood Phe levels per visit are detailed in Table 3.

**Figure 3:**
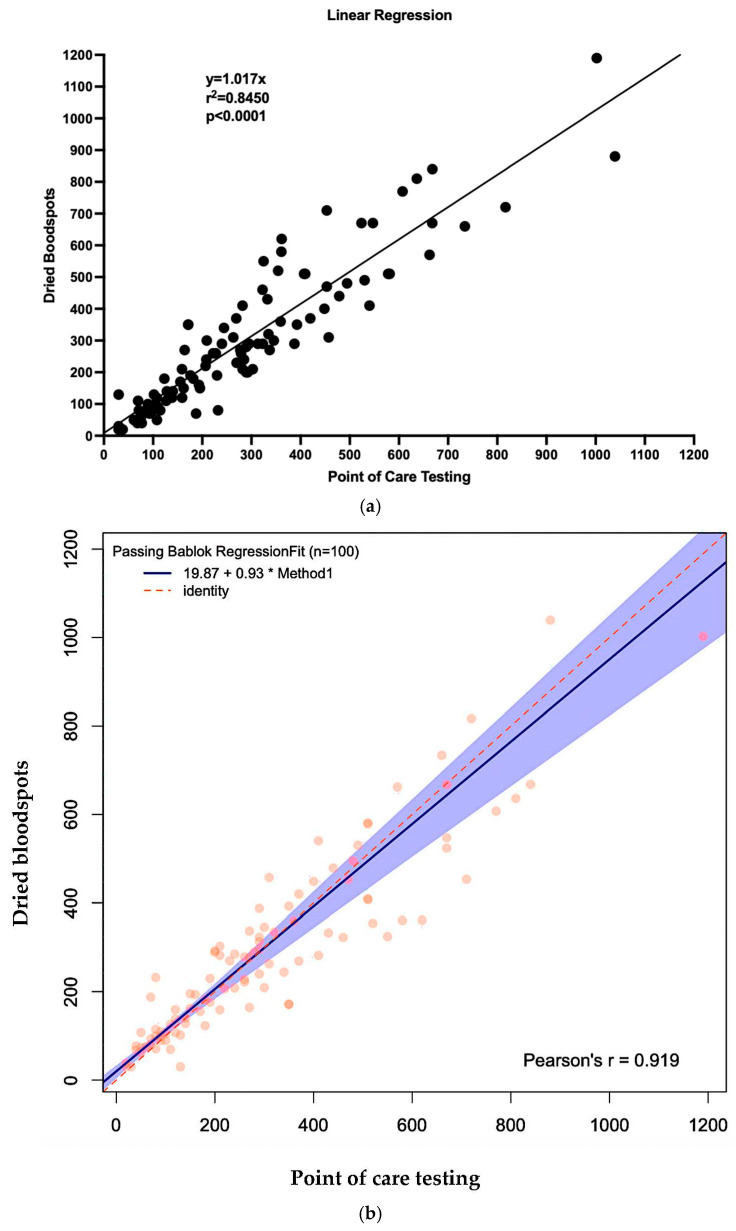
Linear regression curve and Passing Bablok regression comparing POCT to DBS. (**a**) Linear regression. (**b**) Passing Bablok regression.

The curve comparing POCT to DBS was y = 1.017x (R^2^ = 0.8450, *p* < 0.0001), indicating a strong linear correlation between methods (Figure 3a). Application of Passing–Bablok regression, which does not assume a normal distribution and is less affected by outliers, further confirmed the robustness of this relationship, yielding a slope of 0.93 and a correlation coefficient of 0.919 (Figure 3b).

**Figure 4:**
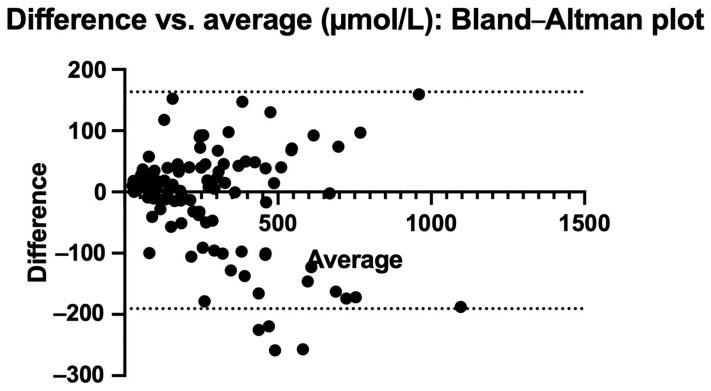
Bland–Altman plot: difference vs. average in µmol/L.

**Figure 5:**
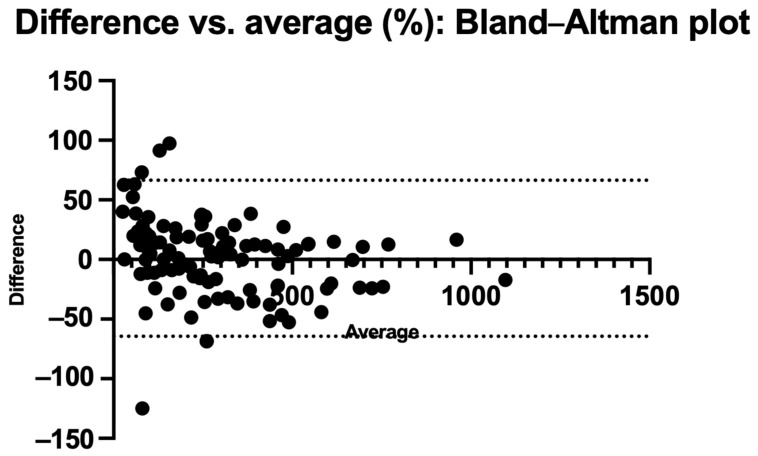
Bland–Altman plot: % difference vs. average.

Figure 4 and Figure 5 also show the Bland–Altman plots illustrating the difference vs. mean of blood Phe levels between methods in µmol/L and % of change, respectively. The Bland–Altman analysis showed only small differences between the two methods, with consistency observed across both low and high Phe concentrations. The mean differences were close to 0%. Outliers and larger discrepancies were primarily attributable to difficulties encountered by caregivers while performing the procedure. These issues are further detailed in the usability data and had been corrected in the majority of participants by the third study visit.

To explore the impact of the calibration strategy, Egoo Phe system results were retrospectively recalculated using a curve derived from the venous plasma reference method. The Egoo Phe system results with this calibration method were 37% higher compared with those obtained with DBS, which is in line with the literature reports. The resulting calibration model is presented in Supplementary Figure S1.

### 3.4. Usability Questionnaire

During each visit, a usability questionnaire was administered by researchers to all participants to evaluate the technique and usability of both POCT and DBS sampling. Supplementary Table S2 presents the complete questionnaire results across visits 1 to 5. Overall, usability scores improved progressively from visits 1 and 2, where initial difficulties reflected a natural learning curve, to visits 3, 4, and 5, where both caregivers and healthcare professionals demonstrated competent technique.

All participants demonstrated a good understanding of English, ensuring consistent interpretation of the questionnaire. The main issues identified across the 100 POCT samples included the following:

- Repeat sampling due to air bubbles in the pipette (n = 17).
- Blood adhering to the inner wall of the Egoo Collect device (n = 5).
- Pipette squeezed more than once, limiting full blood release (n = 2).
- Plasma not successfully separated or visible (n = 2).
- Very low blood Phe levels requiring repeat due to a faulty capsule (n = 2).
- Lid of the Egoo Collect closed prematurely (n = 1).

Most of the procedural issues lessened over time and were resolved by visit 3 as participants gained familiarity with the technique.

### 3.5. End-of-Study Assessment Comparing Both Methods

Although the POCT system required careful handling, all participants expressed a clear preference for the Egoo Phe system over the conventional DBS method. The usability advantages were consistently highlighted in participant feedback, through comments such as “*Better to find out our results quicker*,’ ‘*Sample cannot get lost in the post*,” “*Faster results are very important for adjusting the diet*”, “*Less time consuming, less messy and fewer bruises on the fingers*”, “*You can have the results much faster which is very helpful*” and “*Less blood and faster to do a sample*”. These insights highlight POCT’s potential to improve patient experience and support timely treatment adjustment.

Table 4 reports all the results on the end-of-study assessment comparing DBS and POCT.

### 3.6. Post-Survey Questionnaire

Participants were able to use the POCT system with minimal training in less than 5 min and demonstrated the ability to interpret low, normal, and high Phe levels in patients with PKU, tailored to each child’s treatment targets. Based on the observed performance and participant feedback, researchers felt confident recommending the POCT for home use by parents and caregivers. While overall usability was high, several procedural steps were identified as problematic: collecting the blood sample using the pipette, transferring the blood from the pipette into the Egoo Collect device, and transferring the blood stick from the Egoo Collect into the Phe capsule. These findings suggest that targeted guidance on these specific steps may further enhance ease of use and confidence in home-based monitoring.

Several improvements were suggested by participants to enhance the usability of the POCT system. These included clearer, step-by-step instructions for key stages of the procedure, specifically about filling the pipette, transferring blood into the Egoo Collect device, and inserting the stick into the Phe capsule. Additionally, participants recommended incorporating a colour-change indicator on the stick to improve the visibility of plasma flow, as the current design made it difficult to confirm successful separation (Supplementary Table S3).

## 4. Discussion

This is the first study to evaluate blood Phe levels obtained using the Egoo POCT system (version 2) in comparison with standard DBS sampling using specimens self-collected by participants under home conditions. This study established the reliability and feasibility of the Egoo Phe System in a real-world setting. It differs from a previous report on the Egoo device, which used an earlier version of the machine. We conducted this study under home conditions, focused on caregiver-reported usability, and involved testing across a larger number of samples. In this prospective, observational study, 20 participants were enrolled to evaluate both the analytical differences in Phe levels and the usability of the Egoo Phe System for routine Phe monitoring in PKU. Median blood Phe measured via the Egoo POCT was 4.6% higher than results obtained from DBS samples. This difference may be due to the limitations of the calibration spots. While caregivers were competent at taking finger-pricks for DBS, most had experienced issues with routine DBS monitoring, with 83% reporting having experienced sample rejection within the previous 12 months. In the majority of cases, caregivers posted blood spot samples without allowing prior adequate air-drying. Although initial difficulties with POCT usability were noted, these improved over time, with all caregivers mastering the technique by the third study visit. POCT was consistently described as less time-consuming, easier to perform, and faster in delivering results, factors that may positively influence clinical decision-making and patient engagement as suggested by patients and caregivers by Kuypers et al. [25].

The Egoo Phe system used enzymatic analysis with the aim of producing results similar to venous samples. In our study, the Egoo device was calibrated against standard DBS measurements. The correlation between the two methods was strong (R^2^ = 0.8450, *p* < 0.0001), indicating robust agreement. Although the absolute discrepancy increased at higher blood Phe concentrations, the relative percentage accuracy of the POCT system remained consistent across the measurement range. While DBS remains the standard of care for routine blood Phe monitoring [4], it consistently yields lower concentrations than venous plasma samples [16,20,21,22,23]. Given the limitations of our study design, it remains uncertain which method most accurately reflected true venous Phe levels. Intra-sample variability and lot-to-lot differences are well documented for DBS, with measurement uncertainty typically ranging from 5 to 10% [16,20,21,22,23]. Comparable variability was observed with POCT. To better contextualize POCT results relative to venous sampling, future research should incorporate a controlled, three-way comparison of POCT, DBS, and venous plasma measurements. Additionally, calibration of the Egoo system using DBS versus plasma-derived values warrants further investigation.

Across our study cohort, the majority of participants achieved reliable POCT blood Phe results that correlated well with DBS. However, one individual consistently had lower POCT results, with discrepancies ranging from 31% to 52% below DBS. This outlier prompted targeted intervention, including technique retraining, hydration optimization, and enhanced peripheral blood flow prior to sampling. A possible explanation for this constant difference may be related to higher hematocrit levels [10]. In other participants, any initial variability was attributed to technical and usability challenges, such as insufficient blood volume or suboptimal sample application. These issues were systematically addressed through caregiver education and practice, resulting in improved consistency and alignment between POCT and DBS values.

Inter-laboratory variation in Phe quantification has been reported to reach approximately 20% [32], with assay calibration identified as a key contributor to bias across laboratories [7]. In the UK, most laboratories utilize FIA-MS/MS, which lacks the chromatographic selectivity of liquid chromatography–tandem mass spectrometry (LC-MS/MS). These methodological differences, including calibration standards, extraction protocols, and analytical platforms, may contribute to inter-system discrepancies [24]. To address this variability, several authors have proposed using laboratory-specific correction factors tailored to the calibration procedures and instrumentation in use [16,24], an approach analogous to individualized calibration strategies employed in point-of-care glucose monitoring for diabetes. In this study, the Egoo Phe System was calibrated against the laboratory’s in-house DBS method, providing a consistent reference point for comparative analysis and minimizing external variability. Future models of the Egoo system may incorporate multiple calibration pathways, including the use of Certified Reference Materials (CRMs), to enable direct reporting of plasma Phe concentrations and to support harmonization across analytical platforms to aid robust comparison with external quality assessment (EQA) materials.

The usability of the POCT was assessed in this study. Common difficulties included managing air bubble formation when collecting blood in the pipette and transferring the sample to the Egoo Collect device and applying excessive pressure or prematurely closing the Egoo Collect lid. Users also reported uncertainty in identifying when plasma had fully passed through the collect system due to the absence of a clear visual cue. Notably, technique and confidence improved over time with repeated exposure. In response, the device instructions have undergone revision to address these critical procedural steps, supported by a step-by-step instructional video demonstrating correct technique and highlighting common user error. To enhance clarity and reduce user error, a visual indicator will be integrated into the Egoo Collect device to signal completion of plasma separation. These refinements are intended to improve overall usability and support reliable implementation of the POCT system.

Upon study completion, the majority (90%) of participants reported that the POCT system was easier to use than traditional methods. One caregiver, however, noted that DBS sampling was simpler, citing years of routine practice and familiarity with its technique, which differed from the approach required for POCT. Importantly, no participants reported difficulties in operating the device itself or navigating the Egoo Connect App, indicating that the digital interface had high acceptability and an intuitive design.

At the end of the study, all caregivers expressed a clear preference for the POCT system should it become routinely available, citing reduced burden, faster turnaround, and usability as benefits. Families consistently emphasized the advantage of obtaining results after 29 min, a marked improvement over current practice, in which blood Phe results typically require a minimum of three days and may be delayed by up to a week. Such delays hinder timely treatment adjustments and risk prolonged exposure to elevated Phe levels. In contrast, the POCT system allows for immediate or next-day repeat sampling in cases of technical error or device malfunction (e.g., a faulty capsule or insufficient sample), offering a more responsive and flexible approach to monitoring. This rapid feedback loop was widely viewed as a potential “game changer” in PKU management, with the capacity to reduce neurocognitive risk and support executive function by minimizing the duration of elevated Phe exposure. At the same time, clear instructions and good communication between HCPs and patients/families is needed. Dietary adjustments should only be made after talking to an IMD dietitian to avoid unnecessary adjustments.

POCT offers the potential to lessen the psychological burden associated with waiting for results, empowering families with greater control over blood Phe monitoring and dietary adjustments. This approach may be especially beneficial for families facing social challenges or poor metabolic control, where home-based POCT could serve as a targeted engagement strategy. Currently postal disruptions, bank holidays, and weekend sampling contribute to prolonged turnaround times for blood Phe results, often leading to frustration and even disengagement [25]. POCT could reduce family anxiety when treating newly diagnosed infants, with rapid results enabling healthcare professionals to respond more effectively during the early days of management, improving clinical decision-making and family support. Pregnancy represents another critical window [33] in which POCT could help with monitoring and maternal–fetal outcomes. During pregnancy, it is expected that blood samples be taken at least twice weekly [4], but current postal and laboratory systems often introduce delays that impede timely dietary adjustments, undermining the precision required for optimal metabolic control. Little data is available on blood Phe levels with exercise [34,35,36,37], and POCT would allow for blood Phe levels to be performed during intense exercise, allowing a dietary adjustment and promotion of anabolism on an individual basis. Collectively, these applications highlight the transformative potential of POCT in improving timeliness, reducing anxiety, and supporting more responsive PKU care.

In contrast, the potential costs and reimbursement pathways for POCT devices for blood Phe monitoring are an important concern. It will be essential to rigorously evaluate the cost-effectiveness of POCT devices compared to conventional laboratory-based Phe testing. An economic model is necessary, comparing the costs of the standard system with DBS analysis, including data entry and reporting systems, cost of consumables, equipment maintenance, postal charges, laboratory expenses, personnel, and cost of POCT device. Furthermore, clinical governance and patient safety must be central to POCT implementation. A key concern is that individuals may adjust dietary intake based on single POCT readings, potentially leading to over-restriction or overconsumption of dietary Phe. In its current configuration, the Egoo Phe System does not support automatic transmission of test results to treatment centres. Instead, it will rely on patients manually reporting their blood Phe readings to their clinical team. Manual feedback introduces the risk of delayed, incomplete, or inaccurate communication, placing additional burden on patients and caregivers. Another concern is that there is high dependence on operators (with varying levels of expertise or training), and individuals may fail to detect erroneous results. To ensure the safe and effective implementation of POCT for blood Phe, robust management systems must be established. POCT devices and their users must comply with all relevant regulatory and legal requirements, including device validation, data security, and clinical governance standards. Structured care pathways, underpinned by clinician oversight, are essential to safeguard patient outcomes. This includes comprehensive patient and caregiver training.

Other alternative practical methods of collecting blood Phe samples have been proposed. Carling et al. [32] investigated the use of volumetric blood collection devices (VBCDs) to improve the accuracy and consistency of blood Phe measurements. When paired with a simplified analytical method and standardized calibration, VBCDs demonstrated equivalence with plasma and capillary blood measurements. Notably, a 13% discrepancy observed in DBS was largely recovered (89.4%). Another method, volumetric absorptive microsampling (VAMS), marketed as Mitra^®^ by Neoteryx, uses a hydrophilic polymeric tip to collect precise volumes of blood (10–30 µL) from a capillary finger-prick. The device is self-indicating; its tip turns red when filled, and it is designed with a protective handle to prevent contamination during storage and transport. In a Phase 2 clinical study involving 24 individuals with PKU, 311 matched VAMS and plasma samples were analyzed, demonstrating strong concordance between VAMS-derived and plasma Phe concentrations. These findings support VAMS as a reliable alternative for decentralized Phe monitoring in PKU care [38].

This study has several limitations. A key factor influencing blood Phe results is the usability of the POCT system. Variability in technique can affect outcomes, and the observed differences in blood Phe levels may reflect real-world inconsistencies in sample handling and device operation. A larger sample size would have strengthened the findings. A three-way comparison between venous sampling, DBS, and POCT, conducted under optimal conditions with no technical errors, would provide a more accurate assessment of intrinsic differences between methods. While DBS was used as the reference standard in this study, the comparison is subject to unknown error due to multiple factors that may influence the analytical process. Additionally, our routine monitoring reports Phe levels in increments of 10 μmol/L, whereas the POCT system provides results to the decimal unit. This discrepancy in reporting resolution may contribute to perceived differences in measurements. Due to the measurement uncertainty of the assay being 5–10%, reporting results to the decimal point may be misleading and this is why our DBS results are presented to the nearest 10 and a similar process should be applied to the POCT method when reporting blood Phe results. At very low levels, POCT will only state levels < 30 μmol/L, which may introduce discrepancies compared to DBS results. The Egoo system is currently produced at limited scale. Variability in the manufacturing of devices, Egoo Collect units, pipettes, and capsules may affect overall product quality and consistency, potentially influencing analytical performance. The findings from this study will directly inform improvements to both the user instructions and manufacturing processes of the Egoo Phe system. These refinements will aim to enhance consistency, usability, and analytical reliability across devices and components.

## 5. Conclusions

Blood Phe concentrations obtained using the Egoo POCT system demonstrated close agreement with values derived from DBS analysis, with only a slight positive bias observed. Caregiver proficiency with the POCT system improved over time, and all caregivers reported a clear preference for POCT over DBS sampling. These findings highlight the feasibility and acceptability of POCT as a home-based monitoring tool for individuals with PKU, with the potential to enhance patient autonomy, caregiver engagement, and metabolic control through timely responses to fluctuations in blood Phe levels. Future research should include direct comparisons with venous plasma measurements and formal cost-effectiveness analyses to further establish the clinical utility of the Egoo system in personalized PKU management.

## Figures and Tables

**Table 1:** Difference in results between plasma blood Phe and DBS sampling.

Study	Results
Gregory et al. 2007 [21]	DBS results 15% lower than venous samples.
Groselj et al. 2015 [22]	DBS results 26% lower than venous plasma.
Stroup et al. 2016 [23]	DBS results 28% lower than venous plasma (higher discrepancies with blood Phe levels > 600 µmol/L.
Moat et al. 2020 [16]	DBS results 18 to 28% lower than venous plasma.
van Vliet et al. 2020 [24]	DBS results 4% lower than venous plasma (higher discrepancies in lower levels).
Coene et al. 2021 [20]	DBS results 5% higher than venous samples (variation between −16% and +31%).

**Table 2:** Baseline assement evaluating finger-prick blood test.

Question	Answer	(n, %)
How often do you do finger prick blood spot tests?	More than once a week	(3, 17%)
	Once a week	(12, 67%)
	Once every 2 weeks	(3, 17%)
How easy do you find doing the finger prick blood spot tests?	No problems at all	(12, 67%)
	Usually fine	(4, 22%)
	Usually fine, low blood flow	(2, 11%)
How long does it normally take to do a finger prick blood spot test?	Less than 5 min	(15, 83%)
	5–10 min	(3, 17%)
How long do you allow the finger prick blood spot to dry before putting in the envelope?	I put it straight into the envelope after the test	(7, 39%)
	I wait a few minutes, then put it in the envelope	(7, 39%)
	I wait up to 29 min, then put it in the envelope	(1, 6%)
	I wait up to an hour, then put it in the envelope	(1, 6%)
	I wait up to 2 h, then put it in the envelope	(2, 11%)
When do you usually post the blood spot cards?	On the same day of the test	(14, 78%)
	The next day after the test	(4, 22%)
Have any of the blood spots that you have sent to the laboratory been rejected for analysis in the last 12 months?	Yes	(15, 83%)
	No	(3, 17%)
How many times has this happened?	0	(3, 17%)
	1	(5, 28%)
	2	(6, 33%)
	5	(2, 11%)
	More than 5 times	(2, 11%)
What are the reasons why blood spots have not been analysed? *	Blood spots too small	(12, 67%)
	Taking more than 14 days to arrive to the lab	(6, 33%)
	Incorrect labelling	(1, 6%)
How soon do you normally get your test results back from the dietitian after you have posted them to the hospital laboratory?	1 day after the test	(1, 6%)
	2 days after the test	(4, 22%)
	3 days after the test	(7, 39%)
	5 days after the test	(2, 11%)
	1 week after the test	(4, 22%)

* Parents/caregivers could choose more than 1 option.

**Table 3:** Overall blood Phe levels in each visit.

Visits	POCT	DBS
	Median	Median
	(Range)	(Range)
Visit 1	280	245
	(range: 30–1039)	(range: 30–1190)
Visit 2	308	300
	(range: 61–817)	(range: 50–720)
Visit 3	280	320
	(range: 30–524)	(range: 50–670)
Visit 4	174	200
	(range: 30–734)	(range: 20–670)
Visit 5	209	255
	(range: 68–636)	(range: 40–810)

Abbreviations: POCT—Point-of-care testing; DBS—dried blood spots.

**Table 4:** Results on the end-of-study assessment comparing DBS and POCT.

Question	Answer	(n, %)
How did Egoo Phe System for testing and analysing the blood Phe compare with your usual finger prick blood spot test and sending this to the hospital?	No different	(1, 5%)
	A bit easier	(3, 15%)
	Much easier	(16, 80%)
How did you find downloading and using the Egoo Connect App?	Ok	(3, 15%)
	Easy	(4, 20%)
	Really easy	(13, 65%)
How did you find connecting the Egoo Connect App with the Egoo Analyzer?	Ok	(2, 10%)
	Easy	(5, 25%)
	Really easy	(13, 65%)
How did you find putting the blood sample in the Egoo Collect?	Ok	(1, 5%)
	Easy	(9, 45%)
	Really easy	(10, 50%)
How did you find putting the Egoo Collect into the test capsule?	Difficult	(1, 5%)
	Ok	(2, 10%)
	Easy	(4, 22%)
	Really easy	(13, 65%)
How did you find putting the Egoo test capsule into the Egoo Analyzer?	Ok	(2, 10%)
	Easy	(1, 5%)
	Really easy	(17, 85%)
How did you find scanning the Phe capsules QR code and then receiving the test result on your mobile telephone?	Easy	(1, 5%)
	Really easy	(19, 95%)
How long did it usually take to do the Egoo Phe Test	Less than 5 min	(20, 100%)
What do you think about obtaining a blood Phe result within 29 min in comparison to posting the blood cards to the hospital and taking on average 4 days to receive a blood result?	Really helpful	(20, 100%)
Overall how easy do you find doing the Egoo Phe System test?	Usually fine	(2, 10%)
	No problems at all	(18, 90%)
Which system do you prefer for blood Phe analysis?	Egoo Phe system	(20, 100%)

## Data Availability

The original contributions presented in this study are included in the article/Supplementary Material. Further inquiries can be directed to the corresponding author.

## Supplementary Materials

The following supporting information can be downloaded at https://www.mdpi.com/article/10.3390/nu17233800/s1, Table S1: Percentage of levels in PKU patients below target ranges in each visit; Table S2: Observational questionnaire performed by researchers evaluating POCT and DBS techniques and usability; Table S3: All the results from the post-study questionnaire; Figure S1: Linear regression curve comparing POCT (calibrated for venous method) to DBS.

## References

[1] Blau N., van Spronsen F.J., Levy H.L. (2010). Phenylketonuria. Lancet.

[2] Muntau A.C., Longo N., Ezgu F., Schwartz I.V.D., Lah M., Bratkovic D., Margvelashvili L., Kiykim E., Zori R., Campistol Plana J. (2024). Effects of oral sepiapterin on blood Phe concentration in a broad range of patients with phenylketonuria (APHENITY): Results of an international, phase 3, randomised, double-blind, placebo-controlled trial. Lancet.

[3] Nulmans I., Lequeue S., Desmet L., Neuckermans J., De Kock J. (2025). Current state of the treatment landscape of phenylketonuria. Orphanet J. Rare Dis..

[4] van Wegberg A.M.J., MacDonald A., Ahring K., Bélanger-Quintana A., Beblo S., Blau N., Bosch A.M., Burlina A., Campistol J., Coşkun T. (2025). European guidelines on diagnosis and treatment of phenylketonuria: First revision. Mol. Genet. Metab..

[5] Pinto A., Ahring K., Almeida M.F., Ashmore C., Bélanger-Quintana A., Burlina A., Coşkun T., Daly A., van Dam E., Dursun A. (2024). Blood Phenylalanine Levels in Patients with Phenylketonuria from Europe between 2012 and 2018: Is It a Changing Landscape?. Nutrients.

[6] Baird S., Clinton Frazee C., Garg U. (2022). Quantitation of Phenylalanine in Dried Blood Spot Using Liquid Chromatography Tandem Mass Spectrometry for Monitoring of Patients with Phenylketonuria (PKU). Methods Mol. Biol..

[7] Moat S.J., George R.S., Carling R.S. (2020). Use of Dried Blood Spot Specimens to Monitor Patients with Inherited Metabolic Disorders. Int. J. Neonatal Screen..

[8] Winter T., Lange A., Hannemann A., Nauck M., Müller C. (2018). Contamination of dried blood spots—An underestimated risk in newborn screening. Clin. Chem. Lab. Med..

[9] Chace D.H., Millington D.S., Terada N., Kahler S.G., Roe C.R., Hofman L.F. (1993). Rapid diagnosis of phenylketonuria by quantitative analysis for phenylalanine and tyrosine in neonatal blood spots by tandem mass spectrometry. Clin. Chem..

[10] Carling R.S., Emmett E.C., Moat S.J. (2022). Evaluation of volumetric blood collection devices for the measurement of phenylalanine and tyrosine to monitor patients with phenylketonuria. Clin. Chim. Acta.

[11] la Marca G., Carling R.S., Moat S.J., Yahyaoui R., Ranieri E., Bonham J.R., Schielen P. (2023). Current State and Innovations in Newborn Screening: Continuing to Do Good and Avoid Harm. Int. J. Neonatal Screen..

[12] Lawson A.J., Bernstone L., Hall S.K. (2016). Newborn screening blood spot analysis in the UK: Influence of spot size, punch location and haematocrit. J. Med. Screen..

[13] Wagner M., Tonoli D., Varesio E., Hopfgartner G. (2016). The use of mass spectrometry to analyze dried blood spots. Mass. Spectrom. Rev..

[14] George R.S., Moat S.J. (2016). Effect of Dried Blood Spot Quality on Newborn Screening Analyte Concentrations and Recommendations for Minimum Acceptance Criteria for Sample Analysis. Clin. Chem..

[15] Han J., Higgins R., Lim M.D., Lin K., Yang J., Borchers C.H. (2018). Short-Term Stabilities of 21 Amino Acids in Dried Blood Spots. Clin. Chem..

[16] Moat S.J., Schulenburg-Brand D., Lemonde H., Bonham J.R., Weykamp C.W., Mei J.V., Shortland G.S., Carling R.S. (2020). Performance of laboratory tests used to measure blood phenylalanine for the monitoring of patients with phenylketonuria. J. Inherit. Metab. Dis..

[17] De Kesel P.M., Sadones N., Capiau S., Lambert W.E., Stove C.P. (2013). Hemato-critical issues in quantitative analysis of dried blood spots: Challenges and solutions. Bioanalysis.

[18] Zakaria R., Allen K.J., Koplin J.J., Roche P., Greaves R.F. (2016). Advantages and Challenges of Dried Blood Spot Analysis by Mass Spectrometry Across the Total Testing Process. Ejifcc.

[19] Hogg S.L., Carling R.S., Cantley N.W., Hamilton G., Goddard P., Aitkenhead H., Barski R., Collingwood C., Moat S.J., Kemp H.J. (2023). Cross-sectional audit assessing the quality of dried bloodspot specimens received by UK metabolic biochemistry laboratories for the biochemical monitoring of individuals with Phenylketonuria. Ann. Clin. Biochem..

[20] Coene K.L.M., Timmer C., Goorden S.M.I., Ten Hoedt A.E., Kluijtmans L.A.J., Janssen M.C.H., Rennings A.J.M., Prinsen H., Wamelink M.M.C., Ruijter G.J.G. (2021). Monitoring phenylalanine concentrations in the follow-up of phenylketonuria patients: An inventory of pre-analytical and analytical variation. JIMD Rep..

[21] Gregory C.O., Yu C., Singh R.H. (2007). Blood phenylalanine monitoring for dietary compliance among patients with phenylketonuria: Comparison of methods. Genet. Med..

[22] Groselj U., Murko S., Zerjav Tansek M., Kovac J., Trampus Bakija A., Repic Lampret B., Battelino T. (2015). Comparison of tandem mass spectrometry and amino acid analyzer for phenylalanine and tyrosine monitoring—Implications for clinical management of patients with hyperphenylalaninemia. Clin. Biochem..

[23] Stroup B.M., Held P.K., Williams P., Clayton M.K., Murali S.G., Rice G.M., Ney D.M. (2016). Clinical relevance of the discrepancy in phenylalanine concentrations analyzed using tandem mass spectrometry compared with ion-exchange chromatography in phenylketonuria. Mol. Genet. Metab. Rep..

[24] van Vliet K., van Ginkel W.G., van Dam E., de Blaauw P., Koehorst M., Kingma H.A., van Spronsen F.J., Heiner-Fokkema M.R. (2020). Dried blood spot versus venous blood sampling for phenylalanine and tyrosine. Orphanet J. Rare Dis..

[25] Kuypers A.M., Vliet K.E.-v., MacDonald A., Ahring K., Abeln D., Ford S., Hildebrandt-Karlsen S., van Spronsen F.J., Heiner-Fokkema M.R. (2024). Satisfaction with home blood sampling methods and expectations for future point-of-care testing in phenylketonuria: Perspectives from patients and professionals. Mol. Genet. Metab..

[26] Plebani M., Nichols J.H., Luppa P.B., Greene D., Sciacovelli L., Shaw J., Khan A.I., Carraro P., Freckmann G., Dimech W. (2025). Point-of-care testing: State-of-the art and perspectives. Clin. Chem. Lab. Med..

[27] Point of Care Testing in Community Pharmacies Guidance for Commissioners and Community Pharmacies Delivering NHS Services. https://www.england.nhs.uk/wp-content/uploads/2022/01/B0722-Point-of-Care-Testing-in-Community-Pharmacies-Guide_January-2022.pdf.

[28] Karter A.J., Ackerson L.M., Darbinian J.A., D’Agostino R.B., Ferrara A., Liu J., Selby J.V. (2001). Self-monitoring of blood glucose levels and glycemic control: The Northern California Kaiser Permanente Diabetes registry. Am. J. Med..

[29] Klonoff D.C. (2007). Benefits and limitations of self-monitoring of blood glucose. J. Diabetes Sci. Technol..

[30] Herbert M., Pendyal S., Rairikar M., Halaby C., Benjamin R.W., Kishnani P.S. (2018). Role of continuous glucose monitoring in the management of glycogen storage disorders. J. Inherit. Metab. Dis..

[31] Schoen M.S., Nath S.V., Singh R.H. (2025). Performance of the Egoo test for phenylalanine measurement in females with phenylketonuria. Orphanet J. Rare Dis..

[32] Carling R.S., Barclay Z., Cantley N., Emmett E.C., Hogg S.L., Finezilber Y., Schulenburg-Brand D., Murphy E., Moat S.J. (2023). Investigation of the relationship between phenylalanine in venous plasma and capillary blood using volumetric blood collection devices. JIMD Rep..

[33] Gautiero C., Scala I., Esposito G., Coppola M.R., Cacciapuoti N., Fisco M., Ruoppolo M., Strisciuglio P., Parenti G., Guida B. (2025). The Light and the Dark Side of Maternal PKU: Single-Centre Experience of Dietary Management and Emergency Treatment Protocol of Unplanned Pregnancies. Nutrients.

[34] González-Lamuño D., Morencos C., Arrieta F.J., Venegas E., Vicente-Rodríguez G., Casajús J.A., Couce M.L., Aldámiz-Echevarría L. (2024). Supplementation for Performance and Health in Patients with Phenylketonuria: An Exercise-Based Approach to Improving Dietary Adherence. Nutrients.

[35] Green B. (2025). The exercising patient with phenylketonuria: Considerations and research recommendations. Rare.

[36] Mazzola P.N., Teixeira B.C., Schirmbeck G.H., Reischak-Oliveira A., Derks T.G.J., van Spronsen F.J., Dutra-Filho C.S., Schwartz I.V.D. (2015). Acute exercise in treated phenylketonuria patients: Physical activity and biochemical response. Mol. Genet. Metab. Rep..

[37] Rocha J.C., van Dam E., Ahring K., Almeida M.F., Bélanger-Quintana A., Dokoupil K., Gökmen-Özel H., Robert M., Heidenborg C., Harbage E. (2019). A series of three case reports in patients with phenylketonuria performing regular exercise: First steps in dietary adjustment. J. Pediatr. Endocrinol. Metab..

[38] Gao L., Smith N., Kaushik D., Milner S., Kong R. (2023). Validation and application of volumetric absorptive microsampling (VAMS) dried blood method for phenylalanine measurement in patients with phenylketonuria. Clin. Biochem..

