# Supplementary material for: Point-of-Care Testing in PKU: A New ERA of Blood Phenylalanine Monitoring
Source: Nutrients. 2025 Dec 4;17(23):3800. doi: 10.3390/nu17233800 (PMC12693881; doi:10.3390/nu17233800)
Supplement: Supplementary file 1 [file nutrients-17-03800-s001.zip › nutrients-4008827-supplementary.pdf]

nutrients

MDPI

Supplementary Table S1. Percentage of levels in PKU patients below target ranges in each visit.

Visits	POCT %	DBS %
**Visit 1**		
**<360** µmol/L	61%	61%
**<600** µmol/L	72%	72%
**Visit 2**		
**<360** µmol/L	61%	61%
**<600** µmol/L	89%	94%
**Visit 3**		
**<360** µmol/L	78%	56%
**<600** µmol/L	100%	94%
**Visit 4**	83%	78%
**<360** µmol/L	94%	88%
**<600** µmol/L		
**Visit 5**		
**<360** µmol/L	61%	66%
**<600** µmol/L	88%	78%

Abbreviations: POCT, Point of care testing, DBS, dried blood spots.

Supplementary Table S2. Observational questionnaire performed by researchers evaluating POCT and DBS techniques and usability.

Questions	Visit 1	Visit 2	Visit 3	Visit 4
**- Place the Egoo Analyser on a level, horizontal and stable surface ensuring there is room for the tray to open freely.**	No (45%)<br>Yes (55%)	Yes (100%)	Yes (100%)	Yes (100%)
**- Insert the power cord into the back of the Egoo Device and plug the power supply into a mains power socket**	No (45%)<br>Yes (55%)	Yes (100%)	Yes (100%)	Yes (100%)
**- Open the Egoo Connect App on the Smartphone and in the Home tab press ‘Start Test’ (tray opens on device)**	No (45%)<br>Yes (55%)	Yes (100%)	Yes (100%)	Yes (100%)
**- Check the blue LED “connected” light is showing on the Egoo Analyser**	No (45%)<br>Yes (55%)	Yes (100%)	Yes (100%)	Yes (100%)
**- Place the Egoo capsule in the capsule holder and let the capsule thaw for 10 minutes**	No (45%)<br>Yes (55%)	Yes (100%)	Yes (100%)	Yes (100%)
**- Wash and dry hands**	No (25%)<br>Yes (75%)	Yes (100%)	Yes (100%)	Yes (100%)
	Yes (100%)	Yes (100%)	Yes (100%)	Yes (100%)

**- Remove the safety cap from the lancet, push the tip firmly against the patient's fingertip until the needle is released and pricks the patient's skin**				
**-Wipe off the first drop of blood with tissue paper**	Yes (100%)	Yes (100%)	Yes (100%)	Yes (100%)
**- Massage the finger from its base and along its length to facilitate blood flow Note: do not squeeze the finger, as it can affect the sample quality.**	Yes (100%)	Yes (100%)	Yes (100%)	Yes (100%)
**- Collect blood up to the line on the collection tube**	No (5%)<br>Yes (95%)	No (5%)<br>Yes (95%)	Yes (100%)	Yes (100%)
**- Squeeze the entire blood sample onto the sample collection area of an Egoo Collect**	No (5%)<br>Yes (95%)	No (5%)<br>Yes (95%)	Yes (100%)	Yes (100%)
**- Close the lid and push down the button on the back of the lid completely until you hear a 'click' sound**	No (5%)<br>Yes (85%)<br>Unsure (10%)	Yes (100%)	Yes (100%)	Yes (100%)
**- Wait for the plasma (pale pink) to fill the key shape next to the lid. This should take approximately 30 seconds after closing the lid.**	No (5%)<br>Yes (85%)<br>Unsure (10%)	Yes (100%)	Yes (100%)	Yes (100%)
**- Pull the transfer stick out of the Egoo Collect**	Yes (90%)<br>Unsure (10%)	Yes (100%)	Yes (100%)	Yes (100%)
**- Find the sample inlet on the Egoo Phe Capsule and insert the transfer stick into it. Push it down completely until you hear a 'click' sound.**	Yes (90%)<br>Unsure (10%)	Yes (100%)	Yes (100%)	Yes (100%)
**- Pull the handle up. If the transfer stick is inserted correctly, the handle automatically separates from the transfer stick.**	Yes (90%)<br>Unsure (10%)	Yes (100%)	Yes (100%)	Yes (100%)
**- Place capsule in the open tray of the Egoo analyser and press down until you hear a "click' sound**	Yes (90%)<br>Unsure (10%)	Yes (100%)	Yes (100%)	Yes (100%)
**- Scan the QR code on the capsule using the Egoo Connect App on the smartphone**	Yes (100%)	Yes (100%)	Yes (100%)	Yes (100%)
**- Close the tray by pushing inwards**	Yes (100%)	Yes (100%)	Yes (100%)	Yes (100%)
**- When analysis of the sample is complete (tray automatically opens), remove used capsule and discard**	Yes (100%)	Yes (100%)	Yes (100%)	Yes (100%)
**- Read result from Egoo Connect App**	Yes (100%)	Yes (100%)	Yes (100%)	Yes (100%)
**- Participant knows what the acceptable range of blood Phe is?**	Yes (100%)	Yes (100%)	Yes (100%)	Yes (100%)
**- Participant knows what to do if the result is above the recommended range?**	Yes (100%)	Yes (100%)	Yes (100%)	Yes (100%)
**- Participant knows what to do if the result is below the recommended range?**	Yes (100%)	Yes (100%)	Yes (100%)	Yes (100%)
**- Total score**	21.4 ±3.4	23.9 ±0.4	24 ±0	24 ±0
**- Time to explain procedure**	7.5±3.0 min	5±0 min	5±0 min	5±0 min
**- Was reinstruction required**	NA	No (100%)	No (100%)	No (100%)
**- Time taken to do test (not including waiting for result):**	5	5	5	5
**- Time taken to receive results**	29	29	29	29

Supplementary Table S3. All the results from the post-study questionnaire.

Question	Answer (n, %)
**I (caregiver/parent/ patient) found the Phe machine and blood testing easy to use**	Agree (3, 15%)<br>Strongly Agree (17, 85%)
**Each step in the instructions was clear and easy to follow**	Disagree (1, 5%)<br>Agree (2, 10%)<br>Strongly Agree (17, 85%)
**I am confident that I have done the Phe test correctly**	Agree (3, 15%)<br>Strongly Agree (17, 85%)
**I did not make any mistakes during the test procedures**	Disagree (2, 10%)<br>Agree (5, 25%)<br>Strongly Agree (13, 65%)
**I easily understood the instructions for use**	Disagree (1, 5%)<br>Agree (2, 10%)<br>Strongly Agree (17, 85%)
**I found the guidance clear for each step of the procedure**	Agree (2, 10%)<br>Strongly Agree (18, 90%)
**I understand how to read the test results using the Egoo Connect app**	Agree (5, 25%)<br>Strongly Agree (15, 75%)
**I am confident that the result shown was correct**	Undecided (2, 10%)<br>Agree (4, 20%)<br>Strongly Agree (14, 70%)
**Was the Phe device and its parts easy to hold and operate?**	Undecided (2, 10%)<br>Agree (4, 20%)<br>Strongly Agree (14, 70%)
**The Phe device size and design was convenient for use**	Strongly Agree (20, 100%)

**I did not experience any Phe device malfunction during use**	Undecided (2, 10%)<br>Agree (1, 5%)<br>Strongly Agree (17, 85%)
**No parts of the Phe test stopped working, jammed or got stuck during use**	Agree (13, 15%)<br>Strongly Agree (17, 85%)
**I did not have any technical problems with the Phe device or app**	Strongly Agree (20, 100%)
**No parts of the test caused discomfort injury or concern**	Disagree (1, 5%)<br>Agree (2, 10%)<br>Strongly Agree (17, 85%)
**I did not need to repeat any steps due to mistakes or problems**	Disagree (1, 5%)<br>Undecided (3, 15%)<br>Agree (5, 25%)<br>Strongly Agree (11, 55%)
**I did not have any problems with blood collection (e.g. insufficient sample, excessive bleeding)**	Disagree (2, 10%)<br>Agree (4, 20%)<br>Strongly Agree (14, 70%)
**I was able to see and handle the Phe device, its parts and the smartphone without any trouble during the procedure**	Agree (2, 10%)<br>Strongly Agree (18, 90%)
**All signs in the instructions like listening for a click sound or seeing a colour change in the indicator window, were easy to interpret**	Disagree (1, 5%)<br>Agree (3, 15%)<br>Strongly Agree (16, 80%)

Supplementary Figure S1. Linear regression curve comparing POCT (calibrated for venous method) to DBS.

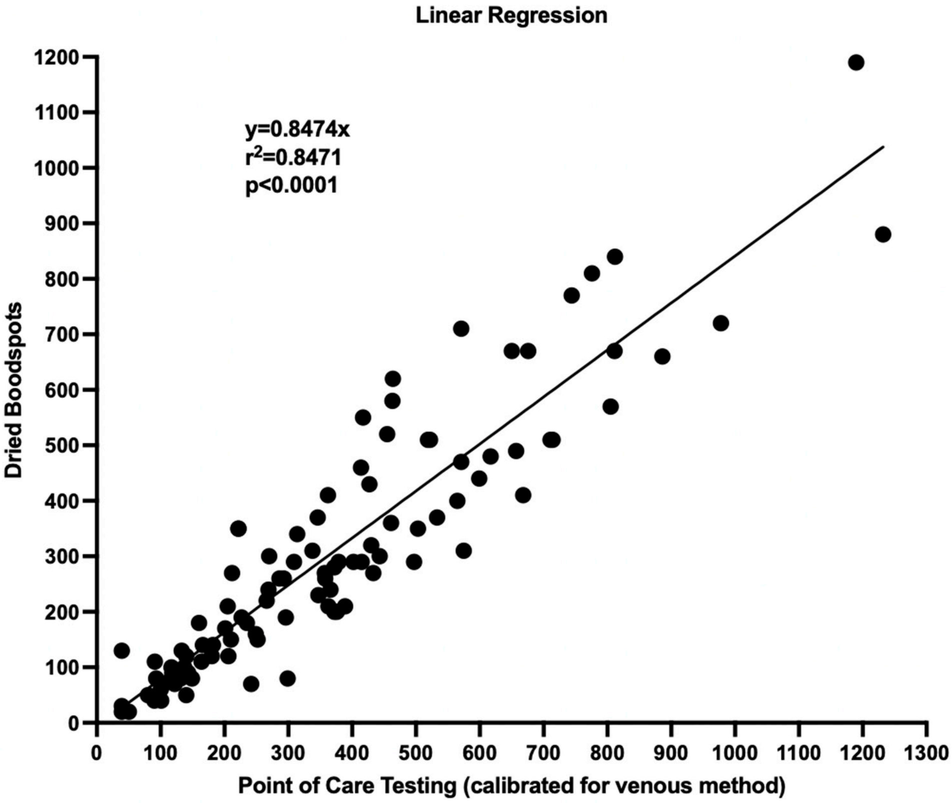
Linear Regression
y=0.8474x
r2=0.8471
p<0.0001
Dried Boodspots
Point of Care Testing (calibrated for venous method)
0
100
200
300
400
500
600
700
800
900
1000
1100
1200
1300
